# Alternative strategies of seed predator escape by early-germinating oaks in Asia and North America

**DOI:** 10.1002/ece3.209

**Published:** 2012-03

**Authors:** Xianfeng Yi, Yueqin Yang, Rachel Curtis, Andrew W Bartlow, Salvatore J Agosta, Michael A Steele

**Affiliations:** 1 College of Agriculture, Henan University of Science and Technology,Luoyang 471003, China; 2 State Key Laboratory of Integrated Pest Management, Institute of Zoology, Chinese Academy of Sciences,Beijing 100101, China; 3 Department of Biology, Wilkes University,Wilkes-Barre, Pennsylvania 18766

**Keywords:** Cotyledonary petioles, hypocotyls, seedling establishment, taproot, white oak

## Abstract

Early germination of white oaks is widely viewed as an evolutionary strategy to escape rodent predation; yet, the mechanism by which this is accomplished is poorly understood. We report that chestnut oak *Quercus montana* (CO) and white oak *Q. alba* (WO) (from North America), and oriental cork oak *Q. variabilis* (OO) and Mongolian oak *Q. mongolica* (MO) (from Asia) can escape predation and successfully establish from only taproots. During germination in autumn, cotyledonary petioles of acorns of CO and WO elongate and push the plumule out of the cotyledons, whereas OO and MO extend only the hypocotyls and retain the plumule within the cotyledons. Experiments showed that the pruned taproots (>6 cm) of CO and WO acorns containing the plumule successfully germinated and survived, and the pruned taproots (≥12 cm) of OO and MO acorns without the plumule successfully regenerated along with the detached acorns, thus producing two seedlings. We argue that these two distinct regeneration morphologies reflect alternative strategies for escaping seed predation.

## Introduction

The evolutionary interactions between plants and animals that disperse their seeds have long been assumed to be a diffuse network of weaker interactions (Janzen 1983; Fleming 1993). However, a growing number of studies show that many plants, especially those dispersed by scatter-hoarding mammals and birds, may exhibit a much closer relationship with their dispersal agents than previously assumed (Steele et al. 2005; Vander Wall 2010). This appears especially true for the oaks (*Quercus*), in which there is strong evidence for a complex ecological and evolutionary relationship between acorns and rodents that act as both seed predators and keystone dispersal agents of these fruits (Steele et al. 2005, 2006).

Much of this rodent–oak relationship centers on specific acorn characteristics and rodent responses (some of which are heritable) to these acorn traits (Steele and Smallwood 2002; Steele et al. 2006). For example, acorns of many white oak species (e.g., subgenera *Quercus* and *Cerris*), in contrast to those of red oaks (subgenus *Lobatae*), exhibit no dormancy and germinate immediately at or even before seed fall, rapidly producing robust taproots (Barnett 1977; Fox 1982; Hadj–Chikh et al. 1996; Steele et al. 2001a, 2006). This strategy is widely regarded as an adaptation to survive seed predation by small rodents (Fox 1982; Hadj–Chikh et al. 1996; Xiao et al. 2010). Although the success of this white oak germination strategy in escaping seed predation has not been fully explored, it is clear that rodent behavior is closely linked to it. In North America (NA), several rodent species selectively store red oak acorns over those of white oak (Smallwood et al. 2001; Steele et al. 2001a, 2007), specifically in response to differences in acorn perishability due to germination schedules (Steele et al. 2001b). Moreover, at least three species of squirrels (*Sciurus carolinensis, S. aureogaster, S. niger*) (Fox 1982; Steele et al. 2001b; Moore 2005; Steele 2008) in NA and two in Asia (*Callosciurus erythraeus* and *Sciurotamias davidianus*) (Xiao et al. 2009, 2010) excise the embryo of white oak acorns before storing them, arresting development to allow long-term storage (Steele et al. 2001a). At least one of these squirrels (*S. carolinesis*) shows a strong innate tendency to store red oak acorns and excise those of white oak (Steele et al. 2006). Elsewhere, one of us has shown that in Asia, Siberian chipmunks (*Tamias sibiricus*) repeatedly use their incisors to prune acorns of *Quercus mongolica* from anchored radicles to arrest or postpone germination, and that other rodents in NA, including the eastern gray squirrel (*S. carolinensis*), similarly prune *Quercus montana* acorns from well-developed radicles (Yang et al. 2011; Yi et al. in prep). One of us also has observed frequent acorn cutting from established acorns of several white oak species by the spotted ground squirrel (*Xerospermophilus spilosoma)* in central Mexico (M. Steele, pers. obs.).

**Figure 1:**
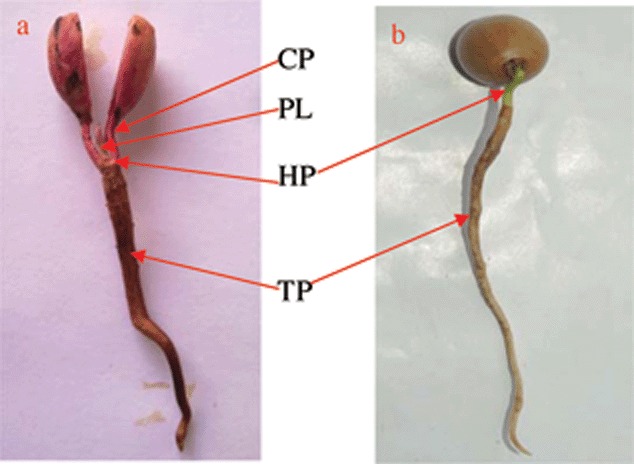
Acorn germination morphologies of two typical white oak species in Asia and North America (NA) in autumn. (a) *Quercus alba*; (b) *Q. variabilis*. Note the cotyledonary petioles (CP), hypocotyls (HP), taproot (TP), and plumule (PL, the embryonic stage of the epicotyl).

Although these observations suggest a strong evolutionary relationship between rodents, especially squirrels and the oaks, we still have a poor understanding of the role of rapid white oak germination in this interaction. Here, we report that taproots pruned from the cotyledons of white oak acorns can regenerate into seedlings and that white oak species from NA and Asia show a strikingly different seed morphology, suggesting alternative strategies to rodent pruning. During autumn germination, cotyledonary petioles in acorns of two NA oak species (chestnut oak *Q. montana*[Sands and Abrams 2009] and white oak *Q. alba*, hereafter CO and WO, respectively) elongate and extend well beyond the apical end of the acorn to the point at which the plumule (embryonic stage of the epicotyl) and radicle diverge, resulting in a separation of the two by 1–2 cm (Fig. 1). In contrast, in two early-germinating oak species in Asia (Oriental cork oak *Q. variabilis* and Mongolian oak *Q. mongolica*, hereafter OO and MO, respectively), autumn germination of acorns is instead characterized by elongation of the hypocotyls (≍1 cm), which results in the plumule and cotyledonary petioles remaining inside the acorn (Fig. 1). The plumule of all four species remains dormant until the following spring. Throughout autumn and early winter, small rodents and jays harvest germinated acorns of white oak species by separating the cotyledons from the anchored taproots (Bossema 1979; Gómez et al. 2003; X. Yi, pers. obs.). For white oaks in NA, rodents prune the cotyledons from the taproots either above or below the plumule (by severing the cotyledons with incisors, X. Yi, pers. obs.), thus generating four types of remnants, that is, pruned acorns with the plumule or without the plumule, and pruned taproots with or without the plumule. For white oaks in Asia, however, the only way for rodents to cut the taproot is to detach the cotyledons along with the plumule because the plumule is always contained in the cotyledons after germination in autumn. Neither Asian nor NA rodents eat the taproots because they are high in fibers, lignins, and cellulose, and are indigestible (Fox 1982; Yang et al. 2011). Although rapid germination and formation of taproots are interpreted as an escape of white oak acorns from seed predation (Fox 1982; Vander Wall 1990), based on these differences in morphology, we suggest that acorns of white oak species from Asia and NA may exhibit different abilities for coping with predation and eventually achieving seedling establishment. Here, we predicted: (1) the pruned taproots and acorns with the plumule of white oaks from NA will regenerate into seedlings; but, the pruned taproots or acorns without the plumule will lose the capacity to produce seedlings; (2) the pruned acorns with the plumule of white oaks from Asia should retain the ability to regenerate into seedlings, whereas taproots without the plumule should not; (3) oak species in NA escape predation by separating the plumule from the cotyledons, whereas oak species in Asia are much less tolerant of radicle pruning.

## Materials and Methods

### Survey of acorn pruning by small rodents

In early October 2009, we tagged 100 germinating OO acorns (20 acorns on each of five 100-m transects) in an oak forest in central China (average elevation 1400 m, 33°45′–33°85′N, 111°75′–112°45′E), by fastening a plastic tag on each of the taproots of the germinating acorns. All acorns had taproots penetrating >10 cm into the soil. Similarly, in early October 2010, we tagged 100 MO acorns (20 per transect) in an oak forest in northeast China (average elevation 750 m, 46°50′–46°59′N, 128°57′–129°17′E), and in November 2010, we tagged 100 CO acorns (20 per transect) in an oak forest in northeast Pennsylvania (see Yi et al. in prep) (average elevation 460 m, 41°05′N, 75°55′W). All three field studies were conducted during years of unusually high acorn mast. In each survey, tagged acorns were checked the following spring and examined for evidence of pruning by rodents and germination success.

### Germination experiments

We collected acorns of WO, OO, and MO from the field and conducted germination experiments on both the acorns and taproots following simulation of taproot pruning. Taproots of each of these three species collected from the field were categorized into six groups according to the length of taproots after pruning: 2, 4, 6, 8, 12, and 16 cm. We imitated rodent pruning by detaching the taproots above or below the plumule from the acorns of WO, and by cutting the taproot at the apical end of the acorn where the radicle emerges for OO and MO acorns. Parallel experiments were conducted on CO as part of a more extensive study on CO pruning (Yi et al. in prep). For CO, one individual pruned acorn or taproot of each length was planted into 20 replicate pots (diameter × height = 15 cm × 20 cm) containing organic composite soil. For WO, 18 pruned acorns or taproots of each length were planted randomly into plastic trays (length × width × height: 4 cm × 4 cm × 10 cm). For OO and MO, 30 pruned acorns and 30 taproots of each length were planted into five replicate pots (diameter × height = 40 cm × 20 cm).

For comparison, we removed the taproots of northern red oak (*Q. rubra*, hereafter RO) acorns following cold stratification (storage at 4°C for at least 90 days) and initial germination. We separated cotyledons (with the plumule) from the taproots (without the plumule) of RO seedlings of various lengths (2, 4, 6, 8, 12 and 16 cm). One individual taproot of each length and cotyledons containing the plumule were planted per pot (diameter × height = 15 cm × 20 cm) and these six treatments were replicated 10 times. We did not test the viability of cotyledons without the plumule and taproots with the plumule, because RO acorns exhibit different germination morphology from WO acorns; that is, the plumule does not emerge until the taproot reaches 8–10 cm (X. Yi, pers. obs.). Therefore, we removed the cotyledons of RO at different stages of seedling development (epicotyl length: 2, 4, 6, 8, 10 cm) to determine whether seedlings can establish without cotyledons. Each of these treatments was replicated 12 times. Pots and trays were kept in the laboratory and cultured at room temperature; acorns and taproots were watered regularly and measured at regular intervals after planting. Seedling survival rates were determined 8 weeks after planting.

## Results

### Field experiments

In the field in China, 76% of the OO acorns and 81% of the MO acorns were either retrieved, eaten, or pruned by small rodents by the following spring (Table 1). Among these acorns sustaining rodent damage, 50.0% (38) of the OO acorns and 33.3% (27) of the MO acorns were pruned by rodents. In northeastern Pennsylvania, 27% of CO acorns sustained damage by rodents (Table 1), possibly because it was the highest acorn crop observed in 12 years (M. Steele, unpubl. data). Of these 27 acorns damaged or removed by rodents, 18.5% (5) and 40.7% (11) were pruned below and above the plumule, respectively, and 40.7% (11) were missing (i.e., removed). We found that 42% (*n*= 16) and 48% (*n*= 13) of pruned taproots of OO and MO regenerated into seedlings, respectively; while 64% (*n*= 7) of CO pruned taproots with the plumule regenerated into seedlings in the field (Table 1).

**Table 1:** Field results of acorn pruning and seedling establishment from pruned taproots of three oak species from North America (NA) and Asia.

	Oak species		
Acorn and taproot types	OO	MO	CO
Total acorns labeled	100	100	100
Acorns pruned above plumule	*	*	11
Acorns pruned below plumule	38	27	5
Acorns missing	38	54	11
Seedling from pruned taproots with plumule	*	*	7
Seedling from pruned taproots without plumule	16	13	0

* Not applicable.

### Germination trials

For both the CO and WO acorns, the pruned taproots containing the plumule successfully germinated regardless of taproot length (Table 2, Fig. 1). Most seedlings survived provided the taproots were ≥ 6 cm in length (Table 2). As expected, the pruned acorns with a plumule germinated into seedlings regardless of the length of taproots (Table 2). Acorns or taproots without the plumule produced only adventitious roots and seedlings never established. Pruned acorns of both CO and MO with the plumule exhibited high germination and seedling survival rates (Table 2). However, contrary to our predictions, pruned taproots without the plumule also regenerated into seedlings provided they were ≥12 cm in length (Table 2, Fig. 1). Thus, the pruned acorns of both OO and MO have the capacity to regularly produce two viable seedlings per acorn: one from the acorn with the plumule and one from taproot without the plumule. Evidently, the hypocotyl tissue contained within the taproot contains sufficient germ tissue to produce a seedling.

**Table 2:** Survival rates of seedlings from pruned taproots and acorns (%). Sample sizes for each treatment were 20 chestnut oaks (CO), 18 white oaks (WO), 30 oriental cork oaks (OO), and 30 Mongolian oaks (MO).

		Taproot length					
Species		2 cm	4 cm	6 cm	8 cm	12 cm	16 cm
CO^1^	Taproots with plumule	0	0	50	75	65	75
	Acorns with plumule	100	100	95	100	85	95
	Taproots without plumule	0	0	0	0	0	0
	Acorns without plumule	0	0	0	0	0	0
WO	Taproots with plumule	0	27.77	44.44	38.89	61.11	77.78
	Acorns with plumule	100	77.78	66.67	100	100	88.89
	Taproots without plumule	0	0	0	0	0	0
	Acorns without plumule	0	0	0	0	0	0
OO	Taproots without plumule	0	0	0	6.67	63.33	76.67
	Acorns with plumule	100	100	93.33	93.33	90	90
MO	Taproots without plumule	0	0	0	3.33	53.33	70
	Acorns with plumule	100	100	100	100	96.67	100
RO	Taproots without plumule	0	0	0	0	0	0
	Acorns with plumule	100	100	100	100	100	100

1 Results for CO were from a more extensive study on CO seed predation (Yi et al. in prep).

Pruned taproots of RO, which consistently had the plumule removed, quickly died regardless of length (Table 2). However, cotyledons with the plumule survived regardless of the length of taproots removed (Table 2). Seedlings of RO without cotyledons survived only when the epicotyl was >6 cm in height (survival rates were 0%, 0%, 41.67%, 83.33%, and 100% for the five treatments, respectively), suggesting an alternative dependence on cotyledonary reserves in white and red oak species.

## Discussion

Our field surveys on acorn pruning, along with those of previous studies on other tree species (Jansen et al. 2006; Cao et al. 2011; Yang et al. 2011) clearly show that rodents regularly prune early germinating seeds. Although we observed a lower frequency of pruning of CO acorns in NA, this most likely resulted from the exceedingly heavy acorn crop in 2010 (M. Steele, unpubl. data). Our results also point to two alternative strategies that white oak species exhibit for potentially dealing with acorn pruning behavior by rodent seed predators. We show that taproots of all four species of oaks (taproots of WO and CO >6 cm containing the plumule and taproots of OO and MO >12 cm without the plumule) already acquired enough reserves to regenerate into seedlings, suggesting considerable resilience for dealing with acorn pruning by rodents. The high capacity of longer taproots to produce seedlings explains the mechanism by which rapid autumn germination allows WO acorns to potentially escape rodent predation (Barnett 1977; Fox 1982). If, in NA, rodents cut and remove acorns following germination, there is a high probability that the established taproot can still result in seedling establishment. In the two Asian species, however, if the acorn is cut and recached, there is potential for both the acorn and the taproot to each produce new seedlings. We argue that regeneration from taproots may be a critical method of seedling establishment in white oak species because their acorns are selectively consumed in autumn over acorns of red oak species due to the low tannin concentrations and high perishability of white oak acorns (Fox 1982; Hadj–Chikh et al. 1996). In contrast, the red oak species, which exhibit delayed germination, may rely more heavily on nutritional reserves in cotyledons for seedling establishment than the white oak species (S. Agosta, unpubl. data). This is supported by the fact that taproots of white oak species can survive without development of the epicotyl (containing only dormant plumules). Hence, this difference in dependence on cotyledon reserves between red and white oak species may in part result from different rodent responses to contrasting germination schedules of the two oak groups (Smallwood et al. 2001).

To our knowledge, this is the first study to verify the capacity of taproots of white oak species to regenerate into seedlings, which we argue represents an evolutionary tactic of their acorns to counter predation by small rodents (Fox 1982). However, we also document different taproot morphologies of white oak species in Asia and NA that we suggest represent two distinct strategies to cope with early predation and pruning by rodents. CO and WO from NA push the plumule out of the acorns to escape predation of the cotyledons, leaving the essential part of oak propagule relatively unharmed, representing an escape strategy from acorn pruning by rodents. In contrast, OO and MO retain the plumule in the cotyledons and extend the hypocotyls on which the taproots without the plumule rely to regenerate into seedlings. This represents a high regeneration strategy (Cao et al. 2011) that can produce a seedling from both the pruned acorn and the taproot.

We also observed an increase in seedling survival with an increase in taproot length (more prominent in Asian oaks), implying a threshold response to nutritional reserves in the pruned taproots. Although taproots without the plumule successfully regenerate into seedlings, white oak species in Asia require a higher threshold level of stored reserves in taproots. These results demonstrate the importance of nutritional reserves in the pruned taproots in supporting seedling establishment of both NA and Asian oaks.

In addition to the species we studied here, several other white oak species (*Q. aliena*, *Q. aliena var. acuteserrata*, *Q. acutissima,* and *Q. liaotungensis*) throughout Asia appear to show a similar germination pattern to OO and MO, in which the plumules are retained in the acorns, but hypocotyls (1–2 cm) are extended out of the cotyledons following autumn germination. Likewise, the formation of cotyledonary petioles (1–3 cm) and the extension of taproot containing the plumule are evident in several other white oaks from NA (*Q. engelmannii*[Engelmann 1880; Coker 1912; Scott 1991; Snow 1991], *Q. macrocarpa*[Green 1959], *Q. glaucoides*, *Q. laeta*, *Q. microphylla*[Yi et al. in prep], *Q. pedunculata*, *Q. platanoides*[*Q. bicolor*], and *Q. austriaca*[Lewis 1911]). Cotyledonary petioles of *Q. virginiana* acorns often reach a length of 5–6 cm and push the plumule into the soil (Lewis 1911), further increasing the probability that the plumule remains attached to the carrot-like taproot when pruned by rodents. Although this pattern of germination of some white oaks (*Q. virginiana* in NA [Lewis 1911]; and possibly *Q. oleoides* in Central America [S. Agosta, pers. obs.] serves as a “sinker” and may provide an advantage for plants in semiarid environments (Lewis 1911; Scott 1991), it also may serve to reduce rodent predation.

Indeed, the geographic ranges over which these two germination strategies occur suggest far more than an exclusive response to environmental conditions. We suggest that these two germination morphologies represent independent strategies for dealing with rodent predation, and that the process of rodent pruning and subsequent establishment of oak seedlings from taproots in NA, and from both taproots and acorns in Asia, is a significant mechanism contributing to oak dispersal and regeneration.
